# Predator interference and complexity–stability in food webs

**DOI:** 10.1038/s41598-022-06524-w

**Published:** 2022-02-14

**Authors:** Akihiko Mougi

**Affiliations:** Institute of Agricultural and Life Sciences, Academic Assembly, Shimane University, 1060 Nishikawatsu-cho, Matsue, 690-8504 Japan

**Keywords:** Ecology, Ecology

## Abstract

It is predicted that ecological communities will become unstable with increasing species numbers and subsequent interspecific interactions; however, this is contrary to how natural ecosystems with diverse species respond to changes in species numbers. This contradiction has steered ecologists toward exploring what underlying processes allow complex communities to stabilize even through varying pressures. In this study, a food web model is used to show an overlooked role of interference among multiple predator species in solving this complexity–stability problem. Predator interference in large communities weakens species interactions due to a reduction in consumption rates by prey-sharing species in the presence of predators in response to territorial and aggressive behavior, thereby playing a key stabilizing role in communities. Especially when interspecific interference is strong and a community has diverse species and dense species interactions, stabilization is likely to work and creates a positive complexity–stability relationship within a community. The clear positive effect of complexity on community stability is not reflected by/intraspecific interference, emphasizing the key role of interspecific interference among multiple predator species in maintaining larger systems.

## Introduction

Natural ecosystems comprise diverse species and their interactions. Although large ecosystems are found in nature^1–4^, May mathematically demonstrated that large ecological communities involving dense species interactions are inherently unstable^5^. This contradiction between nature and mathematics has thus created an ecological mystery^6–8^. In response, considerable attention has been given to solving this so-called complexity–stability paradox^9^. One approach to addressing this paradox is to fill the informational gap between the oversimplification of the mathematical model and complex interactions in nature. Filling this niche could reverse the expected negative complexity–stability relationship into a positive one. Many earlier studies have focused on non-randomized interactions while still housing some temporal and spatial changes^10–12^. However, there are limited network structures that can result in a positive complexity–stability relationship^9^.

May’s model^5^ makes several simplifications regarding network topology and the strengths of species interactions that are contrary to natural ecosystems. Earlier studies tackling the complexity–stability problem have primarily focused on nonrandom network topologies of real food webs because May’s seminal work assumes a random network. Some realistic food web network structures, such as a bias toward donor control^13^, omnivory^14^, compartmentation^15^, and skewness of interaction link distributions^16,17^, can have a stabilizing effect on large food webs. Interaction strength is another key factor that influences community stability because May’s stability criteria^5^ show that strong interaction strengths destabilize systems. Specifically, when species interactions are stronger than intraspecific self-regulation, the system is likely to be unstable. Hence, weaker interactions^18^ or stronger self-regulation^19^ is a reasonable candidate to be a stabilizing factor in complex communities. In fact, in real food webs, the distribution of interaction strengths tends to skew toward weak interaction^20–22^. Weak interaction has played a key role in resolving the complexity–stability debate^7,23^. For example, weak interactions are necessary for allowing multiple interaction types (such as predation, mutualism, and competition) to stabilize larger communities^24–26^. A meta-community, by strengthening self-regulation in each species via migration, may stabilize complex communities^27^. Allometrically linking metabolic and consumption rates to body mass hierarchies by increasing weak interactions with increasing trophic levels also stabilizes complex communities^28^. By creating many weak interactions, adaptive foragers also stabilize complex communities. Cumulatively, these findings suggest that weak interaction is a general factor in stabilizing complex communities^29^.

Another direction that is often overlooked in mathematical models is a non-linear approach to measuring functional responses of species interactions^30^. Functional responses include density-dependent feeding rates—which change in response to abiotic and biotic environmental variation—that play a critical role in understanding consumer–resource interactions and general community dynamics^31,32^. A previous study demonstrated that a type-III functional response promotes an increased number of species interactions; in a type-III response, predation rates increase with increasing prey density and are saturated at a high prey density^33^. Furthermore, this response promotes a level of connectance in the greater food web that increases community stability. A previous study suggested that non-linear functional responses have the potential to resolve the complexity–stability debate^33,34^. A more recent study^30^ used a general model with various functional response types to support this suggestion. Stronger density-dependence in harmful interspecific effects than in that with beneficial ones can create a positive complexity–stability relationship^30^. Although previous studies have shown that functional response variation plays a key role in complexity–stability relationships, they mainly focused on the density-dependent manner of species pairs with direct interaction. However, species interactions are often affected by other species within the same ecological community^35–40^. Thus, the question of how interaction modifications due to indirectly interacting species affect complexity–stability remains unanswered.

Predator interference among multiple predators sharing prey resources is widespread in natural communities^35,41^. It can thus be inferred that the combined effect of multiple predators on shared prey species is a key factor in the resulting community dynamics. A certain level of risk reduction (e.g., lower predation rates of a prey than expected due to emergent impacts of multiple predators) in response to shared prey consumption commonly occurs when predators interfere with the foraging ability^35,41^. Alternatively, such risk reduction can occur via prey’s predator avoidance behavior in response to high predator densities^35,41^. Predator interference is expected to affect community dynamics in numerous ways. Risk reduction results in weak species interactions that ultimately stabilize the community dynamics, while also resulting in competitive exclusion among inferior competitors. In fact, previous studies have argued on both sides of the debate using such models^42,43^. Density-dependence of predators is usually integrated into a prey–predator model as an interference mechanism among same-predator species individuals. In such models, predator interference stabilizes predator–prey dynamics but may also cause an increase in predator extinction when interference is too strong^43,44^. Even when another predator species is incorporated into the predator–prey system, interference among multiple predators has two contrasting effects on predator coexistence: stable coexistence and/or competitive exclusion. The level of interference ultimately depends on the relative strengths of intra- and interspecific interference^42^. In more complex systems with multiple species, predator interference also plays a critical role in community dynamics. Notably, a food web model that included intraspecific interference within same-predator species demonstrated that more connected food webs are indeed stable^45^. Interference among multiple predator species has a stabilizing effect in a complex food web comprising multiple species; however, it also has a negative effect in which multiple alternative equilibria are likely to emerge^42^. Although it has been demonstrated that predator interference can have a stabilizing effect on community dynamics, the models used included interference effects as well as functional responses from multiple prey species^42,45^. This combined approach may not show the true effect of predator interference on community stability. Moreover, it does not address the issue of how interference due to multiple predator species affects the complexity–stability relationship. In the present study, a mathematical food web model and multiple-predator interference approach is adopted to highlight that interspecific interference can result in weak interactions and plays a key role in creating a positive complexity–stability relationship.

The proposed model is based on a food web comprising *N* species, any pair of which is connected with the probability *C* (connectance). Population dynamics are driven by interspecific prey–predator interactions with a non-linear functional response (see “Methods”). A specific functional response, *a*_*ij*_/(1 + *α*_*i*_*X*_*i*_ + Σ_*j*_* β*_*ji*_*X*_*j*_), where *a*_*ij*_ is the consumption rate of resource species *j* by species *i*, *α*_*i*_ is the interference effect of conspecific species to consumption rates, and *β*_*ji*_ is the interference effect of heterospecific species *j* sharing resources to consumption rates, was used. A random food web was assumed to reveal the effect of predator interference with two other network types also tested. Predator interference can occur among conspecific species and/or heterospecific species sharing prey resources. Intraspecific interference decreases consumption rates within a single species, while interspecific interference decreases consumption rates of other species sharing the same resources. The strengths of intra- and interspecific interference are controlled by *α* and *β*, respectively; allowing for an examination of the effects of predator interference on the stability of ecological communities evaluated by community persistence, with the probability that all species persist for a given time (see “Methods” for details).

## Results

**Figure 1:**
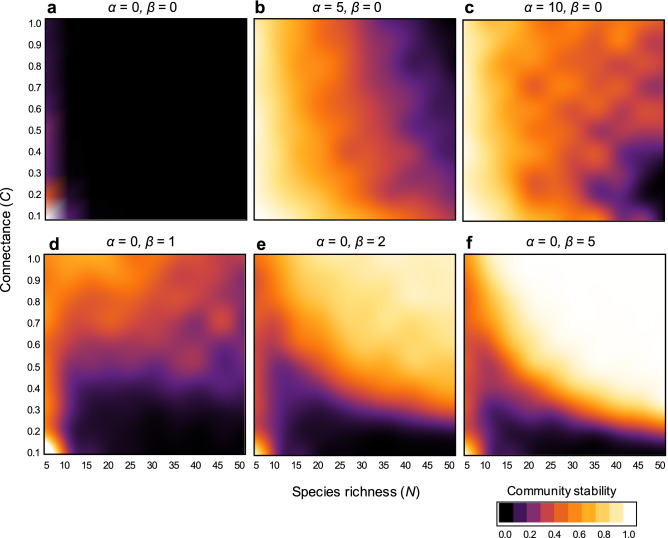
Complexity–stability relationships: (**a**) without interference; (**b**, **c**) with only intraspecific interference; and (**d**–**f**) with only interspecific interference. Color represents community stability defined as community persistence (see the Methods for additional details).

Consider an extreme case without predator interference (*α* = *β* = 0): in a complex community with diverse species and dense interactions, such a system is unlikely to persist, and shows a negative complexity–stability relationship—as shown in previous food web models (Fig. 1a).

**Figure 2:**
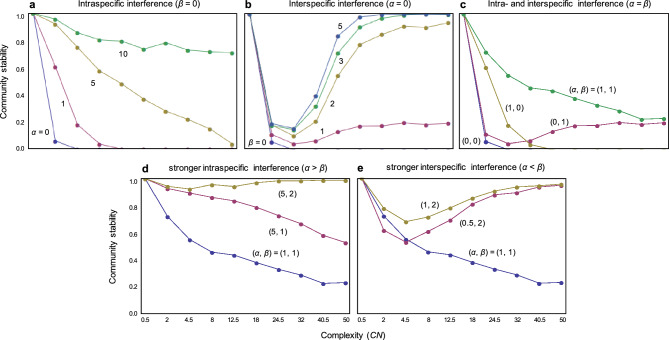
Effects of intra- and interspecific interference on a complexity–stability relationship: (**a**) effects of intraspecific interference (*α* > 0, *β* = 0); (**b**) effects of interspecific interference (*α* = 0,* β* > 0); and (**c**–**e**) effects of intra- and interspecific interference (*α*,* β* > 0). In (**a**, **b**), the strength of either interference type is varied. In (**c**, **e**), the relative strength of the two interference types is different. Horizontal axes refer to complexity, *CN*, which is used as values along the 45-degree line [i.e. (C, N) = (0.1, 5), (0.2, 10), (0.3, 15), (0.4, 20), (0.5, 25), (0.6, 30), (0.7, 35), (0.8, 40), (0.9, 45), and (1.0, 50)]. Community stability is defined as community persistence (see the Methods for additional details).

Here, intraspecific interference is first introduced (*α* > 0,* β* = 0), which increases stability as its strength increases (Figs. 1b, c, 2a). However, intraspecific interference does not change the general negative complexity–stability relationship but it does decrease the strength of this relationship (i.e., the negative slope becomes less steep) (Fig. 2a). More strictly, while species richness cannot increase stability in large multiple-species systems, connectance can increase stability in large multiple-species systems (Fig. 1c).

Next, we consider interspecific interference. To observe the pure effect of interspecific interference on community stability, it was assumed that no intraspecific interference took place in this analysis (*α* = 0,* β* > 0). The results showed that interspecific interference also has a stabilization effect compared with systems without interference (Figs. 1a, d–f, 2b). However, results also revealed three stabilization effects qualitatively different from intraspecific interference. First, interspecific interference has less of an effect on stability in simple systems with an intermediate level of complexity (Fig. 2b), contrary to intraspecific interference (Fig. 2a). In such intermediately complex systems, even if interspecific interference increases, stability remains weak (Fig. 2b), contrary to intraspecific interference (Fig. 2a). Second, interspecific interference has a stronger stabilization effect on more complex systems (Fig. 2b), contrary to intraspecific interference (Fig. 2a). Third, interspecific interference can create positive complexity–stability relationship in both connectance and species richness (Fig. 1e, f), whereas intraspecific interference can create it in only connectance (Fig. 1c). The stabilization effect and positive complexity–stability relationship caused by interspecific interference can be observed regardless of the network type (Fig. S1).

Next, both intra- and interspecific interference (α > 0, β > 0) needs to be considered. The analysis shows that each interference type helps to increase stability among each other (Fig. 2c–e), i.e., one type of interference does not prevent the other from achieving stabilization. Thus, a positive complexity–stability relationship made by interspecific interference can be masked when the interspecific interference is weak and/or intraspecific interference is strong (Fig. 2c and (α, β) = (5, 1) in Fig. 2d). This implies that the positive complexity–stability relationship requires substantially strong interspecific interference (β ≧ 2) and weaker intraspecific interference (Fig. 2e). When both intra- and interspecific interference are strong, the positive complexity–stability relationship becomes weak, but overall stability is always higher ((α, β) = (5, 2) in Fig. 2d). With very large values of both interference effects, complexity has almost no effect on stability and community always maintains perfect persistence (data not shown).

The positive effect of complexity on stability due to interference is also observed when accelerating the density-dependence of interference-squared densities is considered (Fig. S2). Notably, the main result is observed even if a standard type-II functional response corresponding to the densities of multiple prey species is considered (Fig. S3). The multiple prey functional response alone can create a positive complexity–stability relationship (Fig. S3a), as previously reported^34^. Thus, the multiple prey functional response supports a positive complexity–stability relationship even when predator interference is insufficiently strong to create a positive complexity–stability relationship (Fig. S3b). These results suggest that prey handling and predator interference complement and enhance stability for the other and also have the ability to create a positive complexity–stability relationship under varying environmental conditions.

## Discussion

This study served to show that predator interference can make otherwise negative complexity–stability relationships positive. More specifically, interspecific interference among multiple predator species plays a key role in creating a positive complexity effect on community stability. Although both intra- and interspecific interferences have a stabilizing effect on community dynamics, they each play a different role in stabilizing interference effects on system complexity. Intraspecific interference can mitigate destabilization by increasing complexity; however, it maintains May’s negative complexity–stability relationship. In contrast, interspecific interference among multiple predator species can reverse a negative complexity–stability relationship into a positive one. Independently, intra- and interspecific interference have larger stabilizing effects in simple and complex systems, respectively. However, a hybrid approach of both intra- and interspecific interference can have an extremely stabilizing effect on complexity. Such stabilization occurs because each interference level compensates for the other in the system where it functions weakly. These results suggest that interspecific interference has a stronger stabilizing influence in natural systems than previously thought, thus playing an integral role in the maintenance of complex communities.

Intra- and interspecific interference affect community stability in different ways. First, intraspecific interference is independent of the number of interaction links, whereas interspecific interference is not. Furthermore, intraspecific interference does not weaken the interaction strengths of competing species, but does weaken the interaction strength of the species in question. Thus, it acts as a form of self-regulation: if abundance of a species increases, the abundance of the species in question is regulated by that species. However, even in cases where system complexity increases, this self-regulation effect caused by intraspecific interference does not change independently of the amount of interspecific competition, implying that intraspecific interference is unlikely to change the inherent properties of systems or the negative complexity–stability relationship. In contrast to intraspecific interference, interspecific interference weakens the interaction strengths of other competing species. Thus, in the absence of intraspecific interference, interspecific interference can promote competitive exclusion because the superior species is not self-regulated. This is more likely to occur in simpler systems because interspecific interference is unlikely to decrease the consumption rates of each competing species (strong interaction). However, this situation is fully reversed in more complex systems. In larger systems, interspecific interference tends to decrease the consumption rates of each competitor, resulting in weak interactions. This is thought to occur because each species has a small niche space, such as habitat, due to interspecific interference involving multiple species and limited resources. The resulting weaker interactions in complex systems support the positive complexity–stability relationship. It should be noted that a key stabilization factor—namely weak interactions^18,23,46^—results from strong interspecific interferences and/or predator avoidance, which are both common in nature. The different effects of intra- and interspecific interference on the complexity–stability relationship also warrant discussion. Although a stabilizing effect of intraspecific interference was previously reported^45^, the finding was based on empirically estimated body-size structure, which makes it difficult to reveal the pure effect of intraspecific interference on stability. The results of the present study are thus important in that they reveal only the effect of intraspecific interference on stability. A local stability analysis also suggested that intraspecific interference can create a positive complexity–stability relationship^30^ (see SI Appendix). However, in the present analysis, stabilization together with an increase in system size and complexity was not observed, suggesting that a local stability analysis alone cannot capture all features of non-equilibrium community dynamics.

Effort allocation to interspecific interference was not found to qualitatively alter the effects of complexity on stability. In the present model, interspecific interference was found to increase with increasing system complexity. Although predators share more prey species when either the total number of species or connectance increases, this does not necessarily result in more intense interference because of the time allocation to each effort. Additional analysis considers this by assuming that each focal species allocates equal effort to each competitor (i.e., the interspecific interference effects are divided by the number of competitors). Thus, as the number of competitors increases, the magnitude of interspecific interference decreases. As expected, stronger interspecific interference is needed for a positive complexity–stability relationship (Fig. S4a) because effort allocation can reduce the effects of interspecific interference. Although increased species richness in food webs can increase stability, greater connectedness in food webs can decrease stability (Fig. S4b). However, in realistic systems, the number of species interactions or connectance is limited even if the community size becomes large^47–49^. Taken together, stabilization due to interspecific interference can still work with effort allocation. If effort allocation works, the intraspecific interference becomes much weaker than the net effects of interspecific interference (e.g., when *β* is 15, the net magnitude of interspecific interference in communities with 50 species and 0.3 connectance is (by dividing *β* by the mean number of competing species) about 2.35). Using the same calculation, *β* is 7.5 in communities with 20 species and 0.3 connectance. In such communities, if *α* is 8, intraspecific interference is stronger than interspecific interference in complex systems with more than 20 species. This implies that a positive effect of complexity on stability can emerge even when interspecific interference is much weaker than intraspecific interference (Fig. S5). These results suggest several possibilities: (1) Even if intraspecific interference is stronger than interspecific interference, high stability can be maintained in complex communities with many species; (2) Contrary to expectation, interspecific interference may be stronger than intraspecific interference. Niche separation observed in nature may be a consequence of strong interspecific interference, e.g., if interspecific interference is strong, the resources of each species, such as a habitat or territory, are limited; and (3) Interference may not be strong enough to create a clear positive complexity–stability relationship. In such cases, nonlinearity may play a role. A nonlinear functional response of prey use and nonlinear density-dependence of the interference effect can relax the conditions under which interspecific interference creates a positive complexity–stability relationship. In case of nonlinearity of density-dependence of the interference effect, positive complexity–stability can be observed even when the interference effect is moderate. Similarly, in the presence of a nonlinear functional response of prey use, positive complexity–stability may be observed even when the interference effect is moderate. Nonlinearity is a common feature in real systems; thus, a positive complexity–stability relationship may arise from multiple effects of nonlinearity in the functional response of interference under conditions of competition for species and resource use.

To test the theory of the present study, empirical studies comparing the strengths of intra- and interspecific interference need to be conducted. Further, analyzing the relationship between system size—i.e., the number of predators sharing prey resources—and interaction strengths of each species need to be better understood. The present theory can lead to a simple and general coexistence mechanism because of the limitations of behavioral range through predator interference and/or predator avoidance. If multiple species coexist locally, their territories or behavioral ranges are limited, which in turn limits access to prey species, resulting in reduced interaction strength. Contrary to this type of interaction modification—which reduces predation risk—multiple predators can also enhance predation risks^35,41^. This then allows for the assumption that risk enhancement itself should destabilize community dynamics as it increases the strength of interaction. In empirical studies, however, risk enhancement is not likely to be observed in fields—which is contrary to laboratory experiments^41^; suggesting that risk enhancement can occur in narrow spaces where prey is not likely to escape from predators. In contrast, risk reduction is observed more commonly in field studies than risk enhancement^41^. These observations suggest that risk enhancement can—and do—occur in nature, but do so locally and rarely. Further, its destabilization effects may not be strong enough to destroy the stabilization effect in response to risk reduction.

In contrast to an earlier view, interspecific interference may act strongly in nature. In a classical theory, coexistence requires intraspecific competition to be stronger than interspecific competition^50^. However, laboratory experiments and field studies have identified cases in which interspecific interference is equal to or stronger than intraspecific interference in one or both interacting species^51–59^. For example, in land snails (*Euhadra quaestina* vs. *E. peliomphala*)^55^, trout (*Salvelinus leucomaenis* vs. *Salmo trutta*)^60^, and lizards (five sympatric species within the genera *Egernia* and *Eulamprus*)^51^, interspecific interference tends to be stronger than intraspecific interference. In a phytophagous insect community, an examination of 193 pair-wise species interactions found that for approximately 60% of species interspecific interference was equal to (11% of case) or stronger (50% of cases) than intraspecific interference (exploitative and interference competition were equally frequent)^61^. However, such community level examination of intra- versus interspecific interference is extremely limited. To support the findings of the present study, further research on interference competition among multiple species is needed. In addition, interspecific interference may be hidden. If interference is not observed in a focal system, this may be a consequence of interspecific interference. Several review studies have demonstrated that habitat partitioning, time partitioning, and character displacement in competing species^62–64^ are consequences of interference competition. This result suggests that interspecific interference is a major driver of the population dynamics of competing species. The inherent interference effect could be detected through a “removing” experiment involving competing species: if species A begins to use the niche (e.g., habitat, time) of competitor B after the removal of species B, it suggests an inherent interspecific interference effect. By examining the strengths of intra- versus interspecific interference and multispecies functional responses with varying community complexity, we will be able to detect the more realistic effect of interference on community stability.

Detailed analyses of functional response forms have only just begun. A recent study showed that in “one consumer-one resource” systems, a higher order effect (the suite of non-additive effects of interactions between individuals of co-occurring competing species on the fitness of a focal individual) between prey handling and intraspecific interference is not strong^65^, suggesting that a classic Beddington–DeAngelis model^44,66^ with predator interference is a reasonably good approximation for a large number of single-resource consumer-interference data sets. Similarly, in multiple-prey functional responses, a higher order effect between prey handling and other prey handling is likely to be detected; however, a classical multi-prey Holling type-II functional response yielded a good model^65^. Although how the presence of multiple predators and prey individuals alters the aforementioned results is yet to be determined, the results may support that the present model approach is potentially the simplest model to capture complex communities. It may also confirm that positive complexity–stability can be maintained if higher order effects do not act in a way that largely reduces the effects of interference and prey handling. If positive complexity–stability results from the present mechanism, removing species from the equation decreases system complexity and inversely strengthens species interactions, resulting in cascading system destabilization. Moreover, once the system is simplified, it is difficult to rebuild to a larger system again. Such interference between animals is well-known in plant-pollinator mutualistic communities^67^. The results from this and previous studies leaves an unanswered question of what roles animal interferences have in more general ecological communities, including those with various interaction types^24^.

## Methods

Consider a random food web in which pairs of species *i* and *j* (*i*,* j* = 1,…, *N*) are connected by a trophic interaction with a probability of *C*, which is defined as the proportion of realized interaction links *L* in the possible maximum interaction links *L*_*max*_ of a given network model (*L* = *CL*_*max*_). To examine the generalization of the main result, other types of food webs can be tested (Fig. S2). In a cascade model^68^, for each pair of species, *i*, *j* = 1,…, *N* with *i* < *j*, species *i* never consumes species *j*, whereas species *j* may consume species *i.* The maximum link number *L*_max_ is calculated from *N*(*N* − 1)/2 in both random and cascade models. In a bipartite model^69^, no interactions occur within the same trophic levels, and species numbers in each of the two trophic levels are the same, with *L*_max_ = (*N*/2)^2^^2^. The food web model is defined by an ordinary differential equation:

1 $$ \frac{{dX_{i} }}{dt} = (r_{i} - s_{i} X_{i} + \Sigma_{j} M_{ij} X_{j} )X_{i} $$

where *X*_*i*_ is the abundance of species *i*, *r*_*i*_ is the intrinsic rate of change in a species *i*, *s*_*i*_ is the density-dependent self-regulation of species *i*, and *M*_*ij*_ is the interaction coefficient between species *i* and *j*. Interaction coefficients are defined as *M*_*ij*_ = *e*_*ij*_*A*_*ij*_ and *M*_*ji*_ =  − *A*_*ij*_, where *e*_*ij*_ (< 1) is the conversion efficiency.

Next, assume a predator density-dependence functional response, *A*_*ij*_ = *a*_*ij*_/(1 + *α*_*i*_*X*_*i*_ + Σ_*j*_*β*_*ji*_*X*_*j*_)^42,70^, where *a*_*ij*_ is the consumption rate of resource species *j* by species *i*, *α*_*i*_ is the interference effect of conspecific species on the consumption rates, and *β*_*ji*_ is the interference effect of heterospecific species *j* sharing resources on the consumption rates of a focal species *i*. *α*_*i*_ = *α*c and *β*_*ji*_ = *β*c, where c is a constant randomly determined from uniform distribution (0.0 to 1.0), *α* and *β* are control parameters to change the strength of conspecific and heterospecific interference, respectively.

In each iterated simulation, initial species abundance and parameters are randomly selected from the uniform distribution (*X*_*i*_ = 0.01 to 1.0, *r*_*i*_ = 0.05 to 1.0, *a*_*ij*_ = 0 to 0.1, and *e*_*ij*_ = 0.1 to 0.25). The value for *s*_*i*_ was set to a constant of 0.05. Note that in each simulation, each species has a unique set of parameter values and initial abundances. In this model, each species has a positive equilibrium in the absence of interspecific links. If we assume heterotrophic species, an increase in interspecific links decreases the number of heterotrophic species with a nonpotential diet in the food web. Because heterotrophic species with a nonpotential diet cannot persist, increases in interaction links inherently result in positive effects on persistence. To avoid this confounding effect, earlier studies have commonly used a model in which all species persist even in the absence of interaction links^5,8,29,68^. In such models, the community of focus is a local community in which each species uses not only the resources in the focal local community but also external resources from adjacent communities.

Community persistence^29^, which is used as a representative of stability, was then calculated by measuring the fraction of simulation runs in which the entire community persisted (*X*_*i*_ > 10^−5^ for all *i*) after a sufficiently long time period (t = 2 × 10^3^, which corresponded to the time taken for community persistence to reach an asymptote) of 500 runs. The present study did not focus on multiple equilibria. However, because the initial abundances of each species are randomly determined in the present study, the dynamics converge to either equilibrium. If a system with a strong interference effect has multiple attractors, as in a previous model^39^, this may result in higher stability because the system has multiple possible coexistence states.

## Supplementary Information

Supplementary Information.

## Data Availability

All data generated and analyzed during this study are included in the published article.
