# Supplementary material for: Predator interference and complexity–stability in food webs
Source: Sci Rep. 2022 Feb 14;12:2464. doi: 10.1038/s41598-022-06524-w (PMC8844033; doi:10.1038/s41598-022-06524-w)
Supplement: Supplementary file 1 — Supplementary Information. [file 41598_2022_6524_MOESM1_ESM.pdf]

**Supplementary Information**

# Predator interference and complexity-stability in food webs

**Akihiko Mougi**

## Appendix

**Local stability analysis for a functional response with only intraspecific interference (β = 0)**

Consider a case without interspecific interference (β = 0). Then, the system is the following:

$$\frac{dX_i}{dt} = (r_i - s_i X_i + \Sigma_j M_{ij} X_j) X_i \qquad \text{(S-1)}$$

In the following analysis, the parameters and variables are symmetrical $g_i = g$, $r_i = r$, $s_i = s$, $a_i = a$ and $X_i^* = X$, where the asterisk means equilibrium (holding $E(J_{ij}J_{ji}) = 0$). Then, each element of Jacobian matrix, $J$, under an equilibrium is obtained. The diagonal elements are:

$$J_{ii} = -sX - \Sigma g a \alpha X^2/(1+\alpha X)^2. \qquad \text{(S-2)}$$

If the species interaction between $i$ and $j$ occurs, the off-diagonal elements with predator and prey are respectively given by:

$$J_{ij} = gaX/(1+\alpha X) \text{ and } -aX/(1+\alpha X)^2, \qquad \text{(S-3)}$$

otherwise $J_{ij} = 0$.

Here, assume a random large community ($CN$>>1) and g = $1/(1+\alpha X)$ for satisfying $E(J_{ij}) = 0$ for analytical tractability. In this case, a species is expected to interact with $(N-1)C/2$. Then, assuming $N - 1 = N$ for satisfying $CN >> 1$, I obtain

$$E(J_{ii}) = -sX - (CN/2)\{a\alpha X^2/(1+\alpha X)^3\}, \qquad \text{(S-4)}$$

and

$$\mathrm{Var}(J_{ij}) = Ca^2X^2/(1+\alpha X)^4. \qquad \text{(S-5)}$$

For a random community matrix with $CN >> 1$, $E(J_{ij}) = 0$ and $E(J_{ij}J_{ji}) = 0$, the local stability criterion is analytically given by [1]:

$$\sqrt{(N\,\mathrm{Var}(J_{ij}))} < -J_{ii}. \qquad \text{(S-6)}$$

Substituting (S-4) and (S-5) into (S-6), finally I obtain:

$$s > \sqrt{(NC)} * \{a/(1+\alpha X)^2\} * [1 - \sqrt{(NC/2)} * \{\alpha/(1+\alpha X)\}] \qquad \text{(S-7)}$$

When $\alpha = 0$, (S-7) becomes $s > a\sqrt{(NC)}$ which is equal to May's criteria [2]. The inequality (S-7) shows that increasing complexity $CN$ stabilizes the system. This partly supports the stabilization due to an increase in $C$ but cannot explain the tendency of destabilization due to an increase in $N$ in the main text.

## Supplemental figures

**Figure S1**. Effects of network types on a complexity-stability relationship: a) cascade; b) bi-partite; and c) niche. Color represents different values of the strength of interspecific interference; $\alpha = 0$ is assumed. The horizontal axis in (a) is the same as in Figure 2 in the main text, while the axis in (b) has the following values: (C, N) = (0.1, 4), (0.2, 10), (0.3, 16), (0.4, 22), (0.5, 28), (0.6, 34), (0.7, 40), (0.8, 46), and (0.9, 52); and the axis in (c) has: (C, N) = (0.05, 5), (0.1, 10), (0.15, 15), (0.2, 20), (0.25, 25), (0.3, 30), (0.35, 35), (0.4, 40), (0.45, 45), and (0.49, 50). In (a), (b) and (c), the maximum value of $a_{ij}$ is 0.2, 0.1 and 0.1, respectively; $s_i$ was set to a constant of 0.05, 0.05 and 0.1, respectively; and $\alpha = 0$ is assumed. A niche model modified from the original model [3] was used: Species $i$ is assigned with two random values, $n_i$ from a uniform distribution [0,1] and $x_i$ from a Beta distribution with parameters (1, b). Each species has a one-dimensional available niche space with the width, $h_i$, which is given by $n_i x_i$, and the center, $c_i$, which is randomly chosen from a uniform distribution of [$h_i$ /2, $n_i$] when $n_i + h_i /2 \leq 1$, and from [$h_i$ /2, 1 – $h_i$ /2] when $n_i + h_i /2 > 1$. Based on the method by Allesina et al. [3], I did not use the following original rejection criteria: a) a network has connectance level that differs of more than 3% from the one of the empirical food web; b) the network is disconnected; c) two or more species have the same predators and prey. If any of these conditions was met, then the network was rejected. Here, the stochastically builds the networks without considering the above criteria.

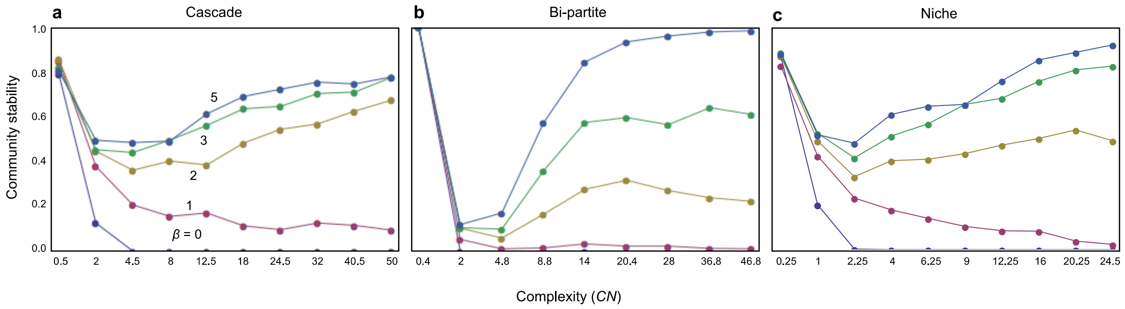

**Figure S2**. Effects of accelerating density dependence of interference on community stability. A squared density effect is assumed – i.e., $A_{ij} = a_{ij}/(1 + \alpha' X_i^2 + \beta' \Sigma_{j \in \text{predators sharing resources}} X_j^2)$. (a) with only intraspecific interference; (b) with only interspecific interference; and (c) with both interference types. Other information is same with Figure 2.

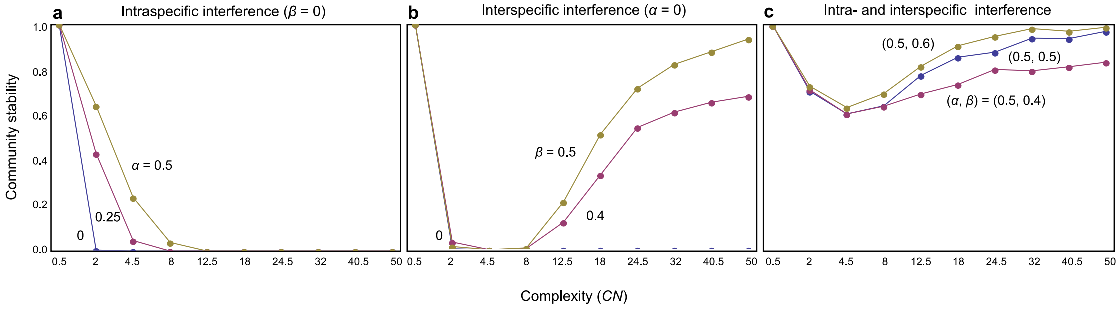

**Figure S3**. Effects of prey handling on community stability. Holling type-II is assumed – i.e., $A_{ij} = a_{ij}/(1 + \gamma'\Sigma_{j\in\text{preys}}X_j + \alpha'X_i + \beta'\Sigma_{j\in\text{predators sharing resources}}X_j)$. $\gamma' = \gamma c$ is the effect of prey handling, $c$ is a constant randomly determined from uniform distribution (0.0 to 1.0), and $\gamma$ represents the control parameters to change the magnitude of prey handling. (a) Without interference; and (b) With interference. Other information is same with Figure 2.

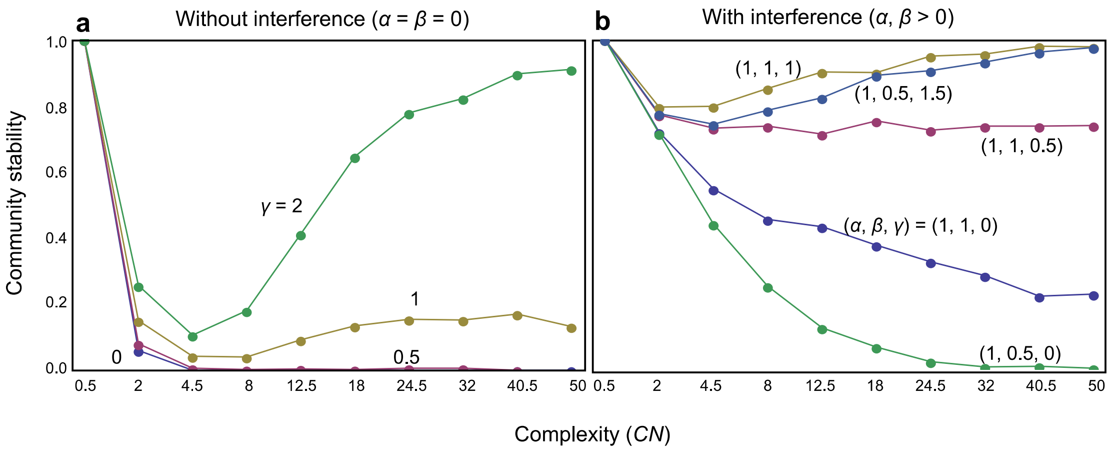

**Figure S4**. Complexity effects on stability in an effort allocation model. (a) Species richness effects; and (b) connectance effects. I assume that each focal species equally allocates the efforts to each competitor (dividing the interspecific interference effects by the number of competitors).The maximum value of $a_{ij}$ is 0.05; $s_i$ was set to a constant of 0.1; and $\alpha = 0$ is assumed. Other information is same with Figure 2.

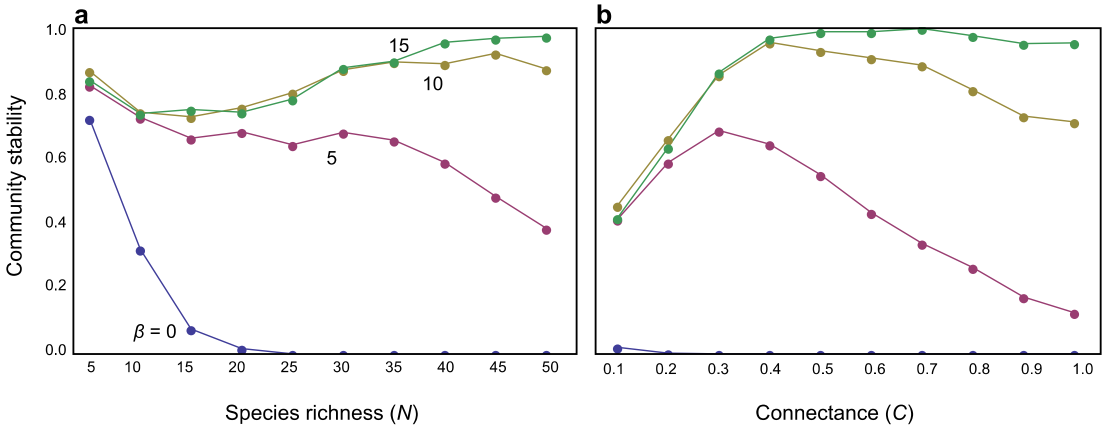

**Figure S5**. Complexity effects on stability in an effort allocation model with both intra- and interspecific interference. (a) Species richness effects; and (b) connectance effects. Although $\alpha > \beta$ is assumed, the net strength of interspecific interference in (a) is 7.5, 5.6, 4.5, 3.5, 3.1, 2.8, and 2.4 with an increase in $N$ (20,…,50), respectively (15 when N < 20); and in (b) 7.5, 4.5, 3.1, 2, 1.6, 1.4, 1.2, 1.1 with an increase in $N$ (20,…,50), respectively (15 when N < 20). Other information is same with Fig. S4.

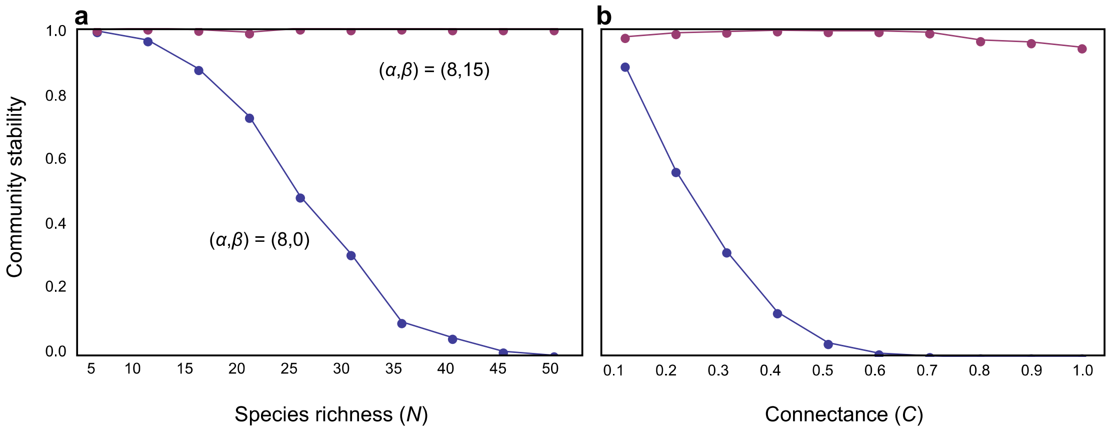


## References

1. Allesina, S. & Tang, S. Stability criteria for complex ecosystems. *Nature* **483**, 205–208 (2012).
2. May, R. M. Will a large complex system be stable? *Nature* **238**, 413–414 (1972).
3. Allesina, S. Alonso, D. & Pascual, M. A general model for food web structure. *Science* **320**, 658–661 (2008).
